# Migration, Agribusiness and Nutritional Status of Children under Five in Northwest Mexico

**DOI:** 10.3390/ijerph9010033

**Published:** 2011-12-28

**Authors:** María-Isabel Ortega, Cecilia Rosales, Jill Guernsey de Zapien, Patricia Aranda, Alejandro Castañeda, Socorro Saucedo, Cecilia Montaño, Alma Contreras

**Affiliations:** 1 División de Nutrición, Centro de Investigación en Alimentación y Desarrollo, A.C., Carretera a La Victoria Km. 0.6, Ejido La Victoria, C.P. 83304, Hermosillo, Sonora, México; Email: coco@ciad.mx (S.S.); cmontano@ciad.mx (C.M.); acontreras@ciad.mx (A.C.); 2 Mel and Enid Zuckerman College of Public Health, University of Arizona, 1295 N. Martin Ave., P.O. Box 245163, Tucson, AZ 85724, USA; Email: crosales@email.arizona.edu (C.R.); dezapien@u.arizona.edu (J.G.Z.); 3 Centro de Estudios en Salud y Sociedad, El Colegio de Sonora, Avenida Obregón No. 54 Col. Centro, C.P. 83000, Hermosillo, Sonora, México; Email: pag@colson.edu; 4 Escuela de Ciencias de la Comunicación, Universidad de Sonora, Blvd. Luis Encinas y Rosales S/N, Col. Centro, C.P. 83000, Hermosillo, Sonora, México; Email: pedroaalejandro@gmail.com

**Keywords:** children under five, migration, health disparities, Mexico

## Abstract

The aim of this study was to examine the nutritional status of children of Mexican migrant worker families under five years of age within the context of global food markets. The sample included 404 children less than five years old from farms and agricultural communities in northwest Mexico. Prevalence of stunting and underweight of children appeared very similar to that of indigenous children from the national sample survey (difference 0.9 and 1.6 percentage points, respectively). Compared to the national sample of Mexican children, stunting and underweight seemed higher in migrant children (difference 17.7 and 4.5 percentage points, respectively), but wasting, an indicator of both chronic and acute undernutrition, appeared to indicate a process of nutritional recuperation. Migrant children living in poverty and suffering from chronic undernutrition, poor performance and scarce education opportunities, can be expected to eventually become agricultural workers with low productivity and poor general health. Consumer’s demands on social and environmental standards of fresh food production in developed countries could be an opportunity to impact the lives of migrant agricultural workers, their families and communities.

## 1. Introduction

Developed countries consumers’ demand for healthy and safe foods have challenged supplying countries and organizations to regulate voluntary standards of social responsibility regarding food production. At first, chemical and microbiological contamination were of concern. Recently, however, health concerns include obesity and chronic diseases and as a consequence an increase in consumption and promotion of fresh organic foods. This has triggered an environment where food production uses biotechnology focused on food safety and environmental care. There is however, a gap on labor related standards and workers living conditions that still need to be addressed [1].

Innovative technologies such as greenhouses and plasticulture production techniques, as well as the increased demand for safe fresh foods, have altered crop cycles. This has increased the demand for field workers and led to longer agricultural periods, requiring new ways of contracting (temporary, intermittent) and forcing enterprises to look for more agricultural temporary workers [1,2,3].

In Northwest Mexico, the supply of regional and local labor is not adequate to meet the demand by agribusiness, influencing enterprises to hire migrant workers from the poorest most southern and indigenous regions of the country [4]. This could be considered a negative impact of global food marketing, since there is an intensive use of labor and environmental resources supporting the cost of new technologies and compliance with international standards on fresh food production. While agribusiness has been successful in the international fresh foods market, the real food producers or agricultural migrant workers have not seen enough improvements in their income and living conditions to assure them and their families a healthy and productive life [1,5]. 

A state survey of agribusiness conducted in the northern state of Sonora, Mexico, in 2001, found that adult agricultural workers were overweight or obese, while children showed stunting and low weight. Low variety dietary patterns, consumption of energy dense low cost foods, and regular periods of food insecurity were related to malnutrition. In addition, parasitic infections were also a frequent source of gastrointestinal diseases among migrant families [6] which indicates poor hygiene of their living environment and housing. Even when tough good agricultural practices (GAP) are being implemented constantly within the harvest, cropping and packing areas, complying with consumer demands on social and environmental standards of fresh food production is still a challenge for public policy in developing countries. [6,7,8].

Barros *et al.* [9] discussed that morbidity, mortality and nutrition of children and mothers from 100 low and middle-income countries have worse prognosis when compared to high–income children and mothers of the same countries. Exposure to poor living conditions during infancy and their consequences such as low birth weight, stunting and impaired cognitive development, have detrimental health effects during adulthood and a significant impact on the human capital of a society. Studies carried out in the UK, Europe, North America and India showed that under nutrition during pregnancy and early life, changes permanently the body structure, physiology and metabolism. Due to lack of plasticity and environmental adaptation capacity, this early body impairment may result in coronary heart disease (CHD) and cerebrovascular disease during adult life [10]. 

In Mexico, The National Survey on Nutrition and Health (ENSANUT) has published that prevalence of stunting, underweight and wasting have decreased in children under five years old from 1988 through 1999 [11]. Sepulveda *et al.* also found a decrease in mortality rates of children less than five years old, from 64 to 23 per 1000 births in 1980 to 2006. Authors attributed this to highly cost-effective strategies that include availability of primary care health services to all households living under the poverty level. However, there are still children in Mexico who are highly vulnerable to the consequences of poverty, such as malnutrition and health related impairments [12]. Migrant worker families laboring in Mexican northwestern states come from the poorest localities within the southern regions in Mexico. These areas include states where the lowest human development indices in the country have been found according to the United Nations Program for Development in 2011. Such states are Chiapas (HDI 0.7395), Guerrero (HDI 0.7594), Puebla (HDI 0.7998), Veracruz (HDI 0.7799), and Oaxaca (HDI 0.7611) [13]. Every year around 200,000 migrant workers come to northern Mexico to work in the vegetable and fruit exporting agribusinesses of the states of Sonora, Sinaloa, Baja California and Chihuahua [14]. 

Migration has been recognized as a poverty alleviating process [1,15], and families migrating from southern to northern Mexico are hopeful, first of all, for the job opportunities that the export agribusiness offers for improving their living conditions. As an indicator of well being of a society, the aim of this study was to analyze the nutritional status of young children from migrant workers families within the context of the global food markets.

## 2. Sample and Methods

The study participants were children under the age of five years, living in farms and agricultural communities from the states of Baja California (three farms and eight nearby communities at San Quintin and Vicente Guerrero Valleys) and Sonora (four rural communities and 12 farms at Costa de Hermosillo, Pesqueira, La Atravesada, Guaymas-Empalme and Caborca Valleys). Sonora and Baja California share a border with the states of Arizona and California in the United States. The study regions are also strongly and dynamically immersed in the international vegetable and fruit market, including fresh products such as tomatoes, Brussels’ sprouts, peppers, zucchinis, cucumbers, strawberries, grapes and watermelon. [16]. Studies at a national level have reported the number of children among migrant agricultural workers in Mexico as 4.7 per family [14]. Lara and De Grammont [17] have estimated the percentage of children 0 to 5 years of age among migrant families in northern Mexico as 11.4%. From these data and the number of agricultural migrant families in Sonora and Baja California reported by Ramirez-Romero *et al*. in 2006 [14] as 2950, we estimated a total of 1585 children 0 to 5 years of age from migrant families living in farms in Sonora and Baja California. We collected data on 596 children 0 to 5 years and discarded the invalid (18) or incomplete data (132), and those data were one of the parents was not a migrant (42). Final sample were 404 children.

### 2.1. Site Characteristics

The sites of worker attraction include those states in northwest Mexico with the highest human development indices within the country (Sonora HDI, 0.8541; Baja California HDI, 0.8557; Chihuahua HDI, 0.8588 and; Sinaloa HDI, 0.8193, according to the United Nations Program for Development 2011 [13]). In contrast, farms hiring migrant workers often lack basic public services, such as water and sewerage systems. Housing is frequently made of cardboard or cement, overcrowded and exposed to extreme temperatures quite different than those of the migrant workers’ home communities. Traditionally, the main states of origin of migrant workers have been Oaxaca, Veracruz, Chiapas and Guerrero, where some Mexican ethnic groups including *Mixteco*, *Zapoteco* and *Triqu*i live. These days, however, migrant workers are also coming from other southern and central Mexican states [18]. Between 25 and 30% of migrant workers in northwest Mexico travel with their families, which include children from infancy to 6 years of age. During working hours most of the children are under the care of older siblings or other migrant women for periods of 10 to 12 hours daily. Some farms have child care facilities, often managed by untrained personnel from the same migrant families. These facilities, however, are inadequate for the high demand of migrant women workers that exists (more than 50%). Some children must thus stay alone or in the company of other young children during working hours. Educational opportunities for these children are scarce and complicated because of their migrant condition [18]. In addition, some studies have shown important nutritional deficiencies among migrant children less than five years old [6,19].

### 2.2. Data Collection

The data for this study were collected during the years of 2004 to 2010. Children’s weight and height were measured by trained staff according to international standards published by the WHO in 1995 [20]. We used digital electronic scales (AND FV-150 K) with 0 to 150 ± 0.05 Kg of capacity, a Holtain Ltd infant and adult stadiometers (146.0 to 92.6 mm and 69.7 to 205.3 cm, respectively). Quality of data was assured in accordance with WHO recommendations regarding spurious data (CDC/WHO) using the Anthro Software, 2010 [21]. Accordingly, valid data were taken regarding the mean of the reference population (WHO, 2006) [22]. For weight for age and height for age (W/A, H/A) data were included between −6.0 and +6.0 Z score. For weight for height (W/H) data included −4.0 and +6.0 Z score. The prevalence of the different forms of undernutrition (stunting, wasting and underweight) were calculated using the baseline of −2 Z scores for each indicator (height-for-age, weight-for-height and weight-for-age) at specific cutoff points for age and sex. 

### 2.3. Data Analysis

Data analysis were performed using SPSS Statistics 17.0 version 17.0.0 Software (SPSS Inc. Chicago, IL, USA). We reported means and standard deviation (*SD*) of the anthropometric indicators as well as prevalence of stunting, underweight and wasting in children of migrant families less than 5 years old. To check the sensitivity of the results (considering the recently change in reference group), the analysis was also carried out using the United States National Center for Health Statistics (NCHS) reference population issued in 1970s [23].

## 3. Results

A total of 404 sampled children belonged to families migrating from the states of Oaxaca (36.9%), Guerrero (27.5%), Veracruz (4.5%), and Chiapas (1.2%). As shown in Table 1 anthropometrical indicators of height for age, weight for age and weight for height of migrant children in this study, are similar to those of indigenous children throughout the country. The three indicators in our sample, however, are worse than those for the national sample of Mexican children, with height for age being the worst, using the WHO 2006 standards [22]. 

**Table 1:** Anthropometric data from children aged less than 5 years in three Mexicans groups.

Anthropometric data	Migrant children (n = 404)	Indigenous Mexican children ^1,2 ^(n = 861)	National survey ^1^ (n = 7707)
Age (mo.)	31.4 ± 15.8	33.2	32.4 ± 27.4
Weight (kg.)	12.1 ± 3.2	12.62	13.1 ± 5.9
Height (cm.)	85.4 ± 11.6	86.64	88.3 ± 23.3
Z score *			
Height-for-age	−1.4 ± 1.2	−1.39	−0.75 ± 2.4
Weight-for-height	0.3 ± 1.0	0.38	0.43 ± 1.8
Weight-for-age	−0.6 ± 1.0	−0.53	−0.13 ± 1.9

* Reference population: WHO 2006 standards [22]; ^1^ Source: [24]; ^2^ Lack of information on ethnicity in the family. Not adjusted standard deviation due to lack of sample size.

**Figure 1:**
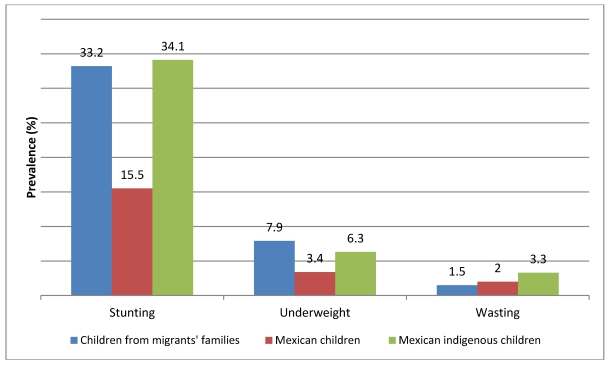
Prevalence of stunting, underweight and wasting in children (<5 years old) from migrants’ families in Northwest Mexico compared with overall Mexican Children and Mexican indigenous children in the 2006 national survey.

Prevalence of stunting, and underweight in migrant children appeared very similar to that in indigenous children from the national sample survey (difference of 0.9 and 1.6 percentage points, respectively), in spite of the fact that among migrant agricultural workers just 40% of families were reported as indigenous [14]. Compared to the national sample of Mexican children, stunting and underweight seemed higher in migrant children (differences of 17.7 and 4.5 percentage points, respectively). Wasting, an indicator of both chronic and acute undernutrition, appeared to indicate a process of nutritional recuperation, since figures are slightly lower than those reported for the national sample and the indigenous children (difference 0.5 and 1.8 percentage points, respectively; Figure 1). 

**Figure 2:**
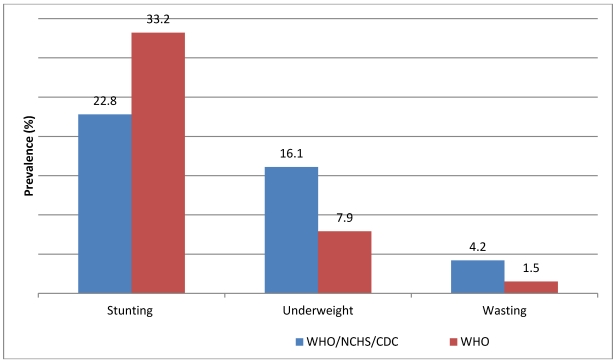
Prevalence of stunting, underweight and wasting in Mexican migrant children (<5 years old) from Northwest Mexico using the WHO/NCHS/CDC (1970’s) and the WHO child growth standards (2006).

Since most of the publications on Mexican childrens’ nutritional status compare data from National Surveys from 1988, 1999 and 2006 [11], Figure 2 shows prevalence of stunting, underweight and wasting in migrant children less than five years old based on the WHO 2006 child growth standards, and the NCHS 1970’s growth standards [23]. Average figures for stunting were 10.4 percentage points higher when the 2006 WHO reference standards [20] were used, while data on underweight and wasting seemed lower. 

Data from the National Survey on Nutrition and Health (ENSANUT) based on the NCHS 1970’s growth standards [11], reported 7.1% of stunting among a general sample of children under five in northern Mexico. Migrant children of the same age have prevalences of stunting 15.7% higher (22.8%). Data for underweight and wasting were 3.9% *versus* 16.1% and 2% *versus* 4.2%, for northern Mexico children and migrant children, respectively (data not shown).

## 4. Discussion

This study found a prevalence of stunting among young children from migrant worker families similar to that reported by the National Survey on Nutrition and Health (ENSANUT) for indigenous children under the age of five in Mexico, and higher than the mean figure for Mexican children in the whole national sample and the sample of children from the northern states, including Sonora and Baja California. The prevalence of wasting (1.5%) was lower in our sample than in the indigenous national sample (3.3%) and similar to the overall Mexican sample, but higher than the general sample of children living in the north of the country [24]. Prevalence of stunting in our sample of Mexican migrant children is quite similar to those reported for children under five in developing countries, especially those classified in the lowest quintile of growth reference standards in the Latin-American and Caribbean populations of the same age. This pattern fulfills the concept of “exclusion” where prevalence of stunting is relatively low in the majority of the population but much higher in a poor and deprived minority portion of the same population [25]. This is one of the few studies in Mexico that presents estimates of under nutrition based on these new standards. 

The development of primary health care systems develop must align with the development stage of a given population. In the case of exclusion, programs targeted to a specific population group, namely the poorest, are encouraged to achieve pro-equity outcomes [25]. Mortality rates of young children have decreased slowly in developing countries, with some exceptions. Within this scenario it is possible that the millennium goals regarding prevalence of under nutrition in young children will not be reached [26]. Since 1982 Mexico has implemented public policies directed to combat malnutrition in young children living under the poverty line and living in marginalized communities [27]. In spite of that, chronic under nutrition is still present in at least 15.5% of the general population and up to 34.1% in under five indigenous children [28]. Therefore, we can infer that at least 1,631,890 children under five will continue within the cycle of poverty and malnutrition under their actual living conditions. This is the case for young children from migrant agricultural workers. 

In 2002 Núñez-Rocha *et al.* reported that having a history of family migration increases 2.61 times the risk of undernutrition in preschool children. These authors support the need for nutritional interventions specifically directed to children who are more vulnerable due to their constant mobility [29]. Although data from studies that evaluate the impact of nutritional and primary health care interventions in Mexican children are encouraging [28], results from our analysis question if public policies are really reaching migrant children. Migrant families must migrate from their impoverished communities to survive, but even when they find jobs, (often seasonal), their children´s inadequate nutritional status is an indicator of poor socioeconomic attainment.

While stunting has decreased in Mexican children under five (form 26.9% in 1988 to 15.5% in 2006), this problem remains pervasive among indigenous children (from 55% in 1988 to 34.1% in 2006) [24]. The impact of programs like Oportunidades (previously PROGRESA) is noteworthy. Implemented in 1997, the program was designed to improve the rate of growth and lower rates of anemia in infants and children in Mexico [30]. The goal of the Oportunidades program is to interrupt the intergenerational cycle of poverty by favoring the development of human capital by providing economic incentives for families to invest in their own future through education, health, and nutrition. The program targets families below poverty guidelines and focuses on keeping children in school. In addition, these programs provide nutritional supplements to children between 6 and 23 months of age as well as underweight children in selected households [31]. However, migrant families moving throughout the country are often not active in these programs making it difficult to impact the nutrition and health of young children’. 

Growth impairment during childhood has several consequences on physical and cognitive development. These consequences include decreased school performance and productivity in general, in addition to being a risk factor for chronic diseases later in life [10,26]. Migrant children living in poverty will have similar labor and productivity outcomes as their parents, thereby perpetuating the poverty and malnutrition cycle. Morover, children with chronic untder nutrition, poor performance and scarce education opportunities, will become agricultural workers with low productivity and poorer overall health [32]. In a study including data from 32,771 adult agricultural migrant workers, Ramírez-Romero *et al.*, found that 75% of them began working in the field during their childhood (45%) and adolesence (28%) [14].

Nutritional and social status of children from migrant agricultural worker families are not aligned with the human rights agenda endorse by The Millenium Development Goals [33]. Indeed, the Rio Political Declaration on Social Determinants of Health, states that the health inequities, referred to as social determinants of health, include early years’ experiences, education, economic status, employment and a livable wage, housing and environment, and effective systems of preventing and treating ill health. In this Conference, government representatives pledged to develop policies that are inclusive and take into account the needs of the entire population, with specific attention to vulnerable groups and high-risk areas. The Conference also urged strengthening and oversight of occupational health and safety protections. The Conference also encourages the public and private sectors to offer healthy working conditions, thus contributing to promoting health for all; in addition to paying special attention to gender-related aspects as well as early child development policies, social and health services [34]. 

Agribusinesses in northwest Mexico are prosperous enterprises in the global food market, but their record of social responsibility toward the wellbeing of their workers or communities surrounding their business, is not altogether realized. The consumer’s insistence for social and environmental standards of fresh food production in developed countries is an opportunity that can impact the lives of migrant agricultural workers, their families and communities [6,7,8].

## 5. Study Limitations

Enrollment of workers and their families in these studies was voluntary and in accordance with the International Review Board policies [35] for reseach with human subjects. Accordingly, the research team observed the non-discriminatory and voluntary participation policies. Sampling these migrant populations is challenging, given they are constantly moving between regions and farms. Moreover, they live and work in private enterprises whose owners are often reluctant to participate in studies investigating the living conditions of their workers. Accounting for these sampling conditions we can assume that there must be a samplig bias that is inherent to a very mobile population. However, we sampled slightly more than a third (37.6) of the total estimated children among migrant families in the study states. In addition, children excluded from the analysis were from different farms and communities, and consequently they did not come from a specific defined group. 

## 6. Conclusions

Children of agricultural migrant workers laboring in the global agribussiness in northwest Mexico, can be considered vulnerable population that continues to be excluded from public policies that protect health or mitigate poverty. Furthermore, if we recognize that migrant families are part of the globalized market for fresh food production involved in improving health in developed countries, the children of these migrant workers are still lacking these same nutritional outcomes, jeopardizing their present and future health status and productivity. 

Public policies regarding nutrition and health of vulnerable Mexican populations must strengthen their efforts to reach poor migrant families, but also Agribusiness owners should contribute to improve the living conditions of workers and their families. Key areas to improve nutrition and health of children are actions of nutritional surveillance and health promotion among migrant families. Adequate day care facilities and trained personal in farms and nearby communities could be the setting for nutritional care of children and health promotion among parents.
